# Antimicrobial Activity of LysX and LysP Endolysins Against *Pseudomonas syringae* pv. *syringae* and *Xanthomonas arboricola* pv. *juglandis*

**DOI:** 10.3390/plants15030431

**Published:** 2026-01-30

**Authors:** Belén Díaz, Pamela Córdova, Alan Zamorano, Melisa Alegría-Arcos, Carlos J. Blondel, Camila Gamboa, Nicola Fiore, Nicolás Tobar, Carolina Ilabaca-Díaz, Assunta Bertaccini, Gastón Higuera

**Affiliations:** 1 Plant Biology and Agro-Food Systems Innovation Laboratory, Institute of Nutrition and Food Technology, University of Chile, Av. El Líbano 5524, Macul, Santiago 7830490, Chile; belen.diaz@inta.uchile.cl (B.D.); pamela.cordova@inta.uchile.cl (P.C.); cilabaca@inta.uchile.cl (C.I.-D.); 2 Laboratory of Plant Virology and Bacteriology, Faculty of Agricultural Sciences, University of Chile, Av. Santa Rosa 11315, La Pintana, Santiago 8820808, Chile; agezac@u.uchile.cl (A.Z.); camila.gamboa@uchile.cl (C.G.); nfiore@uchile.cl (N.F.); 3 Núcleo de Investigación en Data Science (NIDS), Facultad de Ingeniería y Negocios, Universidad de Las Américas, República 71, Piso 3, Santiago 7500658, Chile; malegriaa@udla.cl; 4 Institute of Biomedical Sciences, Faculty of Medicine, Universidad Andres Bello, Santiago 8370071, Chile; carlos.blondel@unab.cl; 5 Molecular and Cell Biology Laboratory, Institute of Nutrition and Food Technology (INTA), University of Chile, Av. El Líbano 5524, Macul, Santiago 7830490, Chile; ntobar@inta.uchile.cl; 6 *Alma Mater Studiorum*—University of Bologna, 40127 Bologna, Italy

**Keywords:** antimicrobial proteins, phytopathogenic bacteria, disease management

## Abstract

*Pseudomonas syringae* pv. *syringae* and *Xanthomonas arboricola* pv. *juglandis* are the causal agents of bacterial canker in cherry and walnut blight, respectively, which cause significant production losses worldwide. These diseases have traditionally been controlled by copper-based agrochemicals and, more recently, antibiotics. However, the prolonged use of these compounds has led to the emergence of resistant bacterial strains. The search for new, efficient, and environmentally friendly biocontrol alternatives has intensified. Phages are promising candidates due to their ability to specifically infect and lyse bacterial pathogens. Endolysin enzymes are responsible for bacterial cell wall degradation, and although they have been extensively studied in medical and veterinary contexts, their application in agriculture remains limited. In this study, 17 putative endolysins were identified from bacteriophages infecting *X. arboricola* pv. *juglandis* and *P. syringae* pv. *syringae*. Based on conserved domain analyses, 12 were classified as glycosidases, four as amidases, and one as an endopeptidase. From these, a recombinant amidase (LysP) and a recombinant glycosidase (LysX) were expressed in *E. coli*, purified, and evaluated as pure enzymes. Both endolysins exhibited significant antimicrobial activity, reducing *P. syringae* pv. *syringae* viability by 62–78.3% and *X. arboricola* pv. *juglandis* viability by 51.5–53.1%, respectively. These findings highlight these recombinant endolysins as promising candidates for the development of biocontrol strategies against bacterial plant pathogens.

## 1. Introduction

In recent decades, the Chilean agricultural industry has experienced significant development that has positioned Chile as one of the largest exporters of raw materials in the southern hemisphere [1,2]. During 2021, fresh and dry fruits dominated world exports [2,3]. In the case of fresh fruits, the cherry emerged as the main exported species, generating revenues of USD 393 million, while the walnut was positioned as the leading species in exports of dried fruits, contributing with USD 215 million [4]. The growth of the agricultural industry faces several challenges in maintaining its global competitive edge, including the phytosanitary status of plants. In this context, despite the efforts of public and private organizations to ensure the improvement and protection of various export plant species, productivity losses associated with crops remain a challenge.

Cherry trees (*Prunus avium* L.) are infected by phytopathogenic microorganisms like fungi, pseudofungi, viruses, bacteria, including phytoplasmas, and nematodes [5]. The main cause of production losses is the bacterial canker caused by *Pseudomonas syringae* pv. *syringae*. Bacterial canker usually affects young trees, and it has been reported that in extreme cases it could cause the death of over 50% of the trees in an orchard [6]. In the case of adult trees, defense responses are triggered by the secretion of dark gum. These secretions, known as gummosis, usually manifest around areas of necrosis with a distinctive fermentation odor. These secretions usually manifest around areas of necrosis or cankers in the wood [5,7]. However, the progression of the infection generates a decrease in the quality of the fruit and, over time, death of the branch or, in extreme cases, the death of the entire tree. Walnut trees (*Juglans regia* L.) are infected by various phytopathogenic microorganisms, such as fungi, nematodes, viruses and bacteria [8]. However, the walnut blight caused by *Xanthomonas arboricola* pv. *juglandis* is the most relevant bacterial disease due to the production losses associated with it [9], causing production losses up to 50% [10]. The infection enters the walnut tree through natural openings (stomata and lenticels) and wounds [8]. Additionally, in mature nuts, the infection can discolor the shell and pulp of the nuts, leading to their premature fall.

Currently, the main strategy to contain the diseases caused by phytopathogenic bacteria is based on cultural management and the use of copper-containing agrochemicals or antibiotics [11,12]. The use of these agrochemicals over the last decades has led to a reduction in the effectiveness of treatments, due to the selection of bacterial strains resistant to copper, antibiotics or both [13,14,15]. The loss of effectiveness of current treatments has driven new research looking for alternatives to control these phytopathogenic microorganisms. Numerous studies have reported a wide variety of organisms and biological compounds that demonstrate antimicrobial capacity [16,17,18], with bacteriophages standing out among them. Due to their outstanding lysis capacity, bacteriophages are recognized as ideal candidates for various strategies to control pathogenic bacteria (phage therapy) in several fields, including human medicine, veterinary and agricultural industries [19,20]. Despite this, bacteriophages have shown several disadvantages. The most relevant is the development of bacterial resistance to bacteriophages, which can be acquired by bacteria through different mechanisms [21].

Due to advances in research and increased knowledge of bacteriophage biology, phage proteins known as endolysins have been developed as potential candidates for biocontrol strategies. Endolysins are proteins produced by bacteriophages that destroy the bacterial envelope during the final stage of the phage replication cycle, leading to cell lysis [21]. This mechanism involves the hydrolysis of peptidoglycan in the bacterial cell wall, resulting in the release of the bacterial cell’s internal contents [22]. Due to their ability to degrade peptidoglycan, endolysins are considered excellent therapeutic agents, with studies demonstrating their potential in human and animal medical applications to control bacterial infections, including those caused by antibiotic-resistant strains [23,24,25,26,27,28,29]. However, there is limited research in agriculture, where phage endolysins could serve as potential candidates for biocontrol strategies against pathogenic bacteria that infect crops of economic importance. In the present study, it is reported for the first time the identification, recombinant expression, and bactericidal evaluation of endolysins derived from bacteriophages infecting the phytopathogenic bacteria *X. arboricola* pv. *juglandis* and *P. syringae* pv. *syringae*. Within these phages, three families of endolysins were identified: 12 classified as glycosidases, four as amidases, and one as an endopeptidase. The *in vitro* antimicrobial activity of two selected enzymes (one amidase and one glycosidase) against *P. syringae* pv. *syringae* and *X. arboricola* pv.* juglandis* was then evaluated. The tested endolysins were found to reduce the viability of *P. syringae* pv. *syringae* in the range of 62–78.3%, and that of *X. arboricola* pv.* juglandis* by 51.5–53.1%, respectively.

## 2. Results

### 2.1. Identification and Characterization of Genes Encoding Endolysins

Genomic analysis of 16 bacteriophages led to the identification of 25 putative endolysin-encoding genes (Table 1). Sequence analysis revealed that eight of these genes could not be functionally assigned; therefore, subsequent analyses focused on the remaining 17 genes. Among them, 12 encoded endolysins belong to the glycosidase family, four to the amidase family, and one to the endopeptidase family. The endolysins were grouped based on their catalytic domains, reflecting the diversity of peptidoglycan-binding and cleavage mechanisms (amidase, glycosidase, and endopeptidase) present in the analyzed phage genomes (Supplementary Table S1).

Among the detected endolysins, two with potential antimicrobial activity were selected based on selection criteria, including an identity value ≥ 75% and coverage ≥ 80% [30]; the presence of specific lytic domains was confirmed using InterPro v97.0 [31], which identified either lysozyme domains (IPR023346) or N-acetylmuramoyl-L-alanine amidase domains (IPR036505). Accordingly, LysX, a glucosidase derived from phage Xaj4.1, and LysP, an amidase derived from phage Pss63, were selected for further analysis.

**Figure 1:**
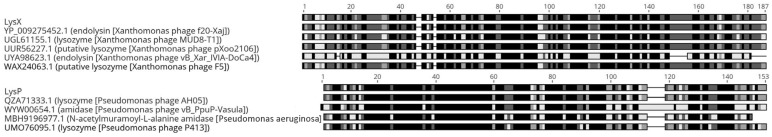
Amino acid sequence similarity between the identified endolysins LysX (**top**) and LysP (**bottom**) and the homologous protein identified by BLASTP (version 2.15.0). Each row corresponds to a protein sequence, and the horizontal axis indicates the amino acid position. Shaded blocks represent the degree of identity between sequences: black indicates complete identity, gray indicates different levels of similarity, and white represents non-conserved regions or gaps. Continuous dark blocks highlight highly conserved regions among the analyzed endolysins.

The endolysin LysX showed 100% identity and 100% coverage in its amino acid sequence with the glucosidase family of *Xanthomonas* phage f20-Xaj, according to the BLASTP alignment. In addition, LysX shared sequence identity with other phages, including *Xanthomonas* phage F5 (97%), *Xanthomonas* phage pXoo2106 (95%), *Xanthomonas* phage MUD8-T1 (81%), and *Xanthomonas* phage vB_Xar_IVIA-DoCa4 (46%) (Figure 1, top panel). LysX has a length of 185 amino acids, corresponding to an approximate molecular mass of 21.8 kDa. *In silico* analysis predicted the presence of an endolysin_R21-like domain (residues 27–169), with putative catalytic residues located at amino acids E(37), D(46), and T(52).

Similarly, the LysP sequence showed 100% identity and 100% coverage with the endolysin of *Pseudomonas* phage AH05. In addition, LysP displayed sequence similarity with several other endolysins, including those from *Pseudomonas* phage Pf1 ERZ-2017 (88%), *Pseudomonas* phage P413 (82%), *Pseudomonas aeruginosa* (65%), and *Pseudomonas* phage vB_PpuP-Vasula (64%) (Figure 1, bottom panel). LysP has a length of 146 amino acids, with a theoretical molecular mass of 17.6 kDa. In addition, analysis indicated the presence of an amidase_2 domain (residues 13–133), including predicted Zn-binding, substrate-binding, and amidase catalytic sites.

**Figure 2:**
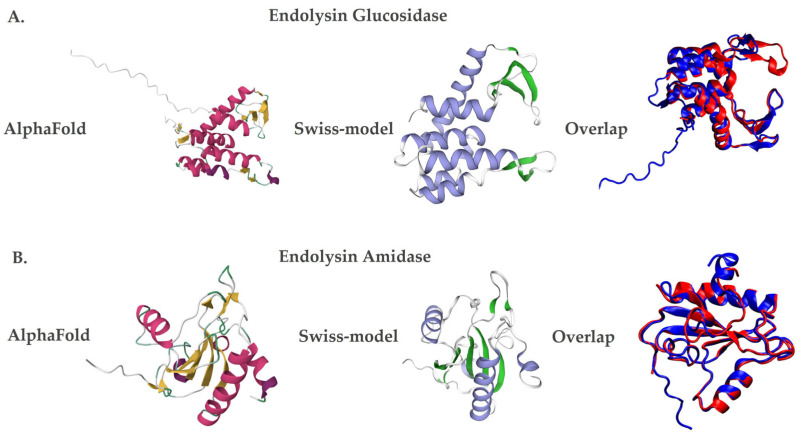
Three-dimensional structural models of the endolysins LysX and LysP. Protein structures were predicted using AlphaFold (left panels; red and yellow color scheme) and Swiss-Model (middle panels; violet and green color scheme). Structural superposition of both models is shown in the right panels (blue for AlphaFold and red for Swiss-Model). (**A**) LysX, a glucosidase-like endolysin derived from *X. arboricola* pv. *juglandis* phage 4.1. (**B**) LysP, an amidase-like endolysin derived from *P. syringae* pv. *syringae* phage 63.

Additionally, the three-dimensional structural models of the endolysins LysX and LysP were generated using AlphaFold [32], based on artificial intelligence, and Swiss-Model [33], through homology. Both methods showed similar structures, but with differences in the number of secondary structures. In the case of LysX, the AlphaFold [32] predicted seven alpha helices and eight beta sheets, while the Swiss-Model [33] predicted six alpha helices and five beta sheets (Figure 2A). Similarly, for LysP, the AlphaFold showed five alpha helices and ten beta sheets, while Swiss-Model identified four alpha helices and seven beta sheets (Figure 2B).

**Figure 3:**
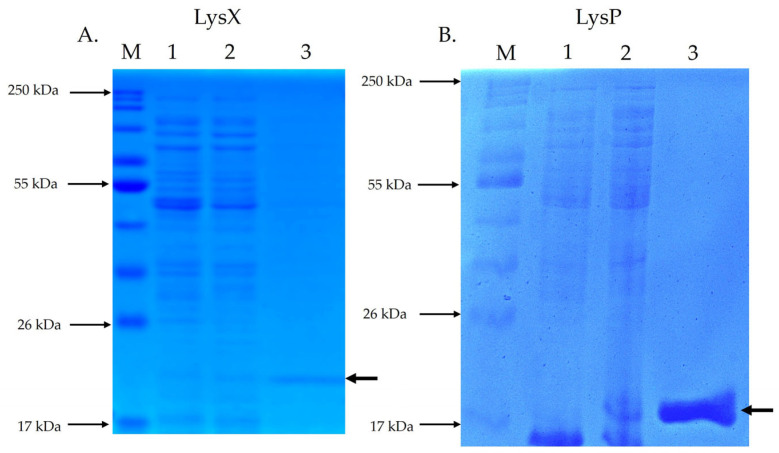
SDS-PAGE analysis of recombinant endolysins LysX and LysP. The electrophoretic profile of total and purified proteins was evaluated by SDS-PAGE. M: molecular size marker; Lane 1: total protein without IPTG induction; Lane 2: total protein with IPTG induction; Lane 3: purified endolysin. The arrow indicates the position of the recombinant endolysin. (**A**) shows the SDS-PAGE of LysX (21.8 kDa), while (**B**) shows the SDS-PAGE of LysP (17.6 kDa).

SDS-PAGE analysis of endolysin LysX (Figure 3A) revealed a protein band with electrophoretic homogeneity at the expected size of 21.8 kDa. Similarly, SDS-PAGE analysis of endolysin LysP (Figure 3B) showed a protein band with electrophoretic homogeneity at the expected size of 17.6 kDa.

### 2.2. Antimicrobial Activity of Recombinant LysX and LysP Proteins

**Figure 4:**
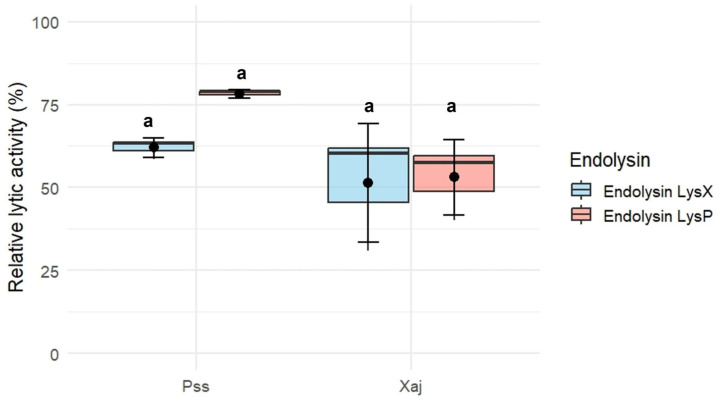
Lytic activity against *P. syringae* pv.* syringae* (Pss) strain S2 and *X. arboricola* pv. *juglandis* (Xaj) strain 66a of recombinant endolysin LysX and LysP (10 µg/mL). Error bars represent the standard error of the mean (SEM, n = 3). Different letters indicate statistically significant differences (*p* < 0.05), whereas shared letters (*e.g.*, “a”) denote groups that are not significantly different, as determined by the Kruskal–Wallis test followed by Dunn’s post hoc test.

The endolysins LysX and LysP exhibited lytic activity against *P. syringae* pv. *syringae* S2 and *X. arboricola* pv. *juglandis* 66a (Figure 4). Using a concentration of 10 µg/mL for each endolysin, the relative lytic activity of LysX was 62 ± 2.99% against *P. syringae* pv. *syringae* S2 and 51.5 ± 17.9% against *X. arboricola* pv. *juglandis* 66a. In the case of LysP, the relative lytic activity was 78.3 ± 1.26% against *P. syringae* pv. *syringae* S2 and 53.1 ± 11.3% against *X. arboricola* pv. *juglandis* 66a.

### 2.3. Characterization of Recombinant Endolysin LysX and LysP

**Figure 5:**
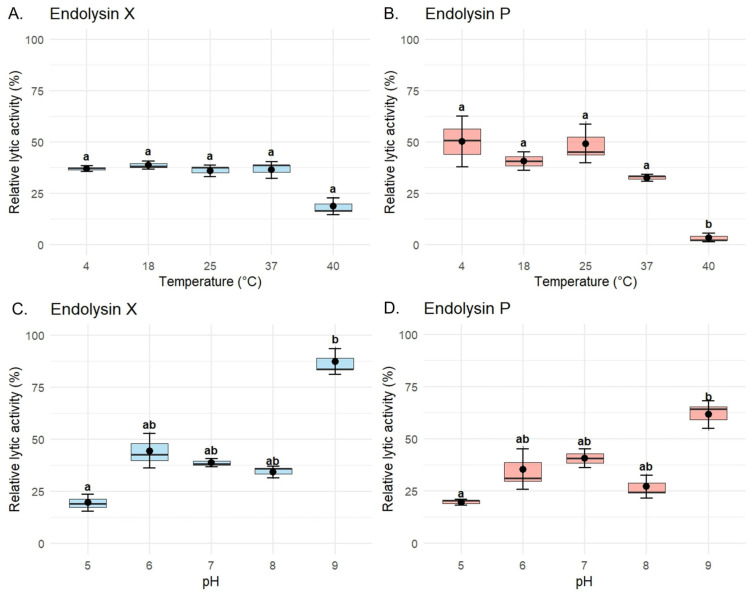
Thermostability and pH dependence of the lytic activity of recombinant endolysins LysX and LysP. Lytic activity assays for characterization were performed against *P. syringae* pv. *syringae* strain P63 using a final concentration of 10 µg/mL of purified enzyme. (**A**,**B**) Lytic activity profiles of LysX (**A**) and LysP (**B**) after incubation at different temperatures (4, 18, 25, 37, and 40 °C). (**C**,**D**) Lytic activity profiles of LysX (**C**) and LysP (**D**) under varying pH conditions (pH 5.0, 6.0, 7.0, 8.0, and 9.0). Normality and homogeneity of variances were evaluated using the Shapiro–Wilk and Levene’s tests, respectively. For statistically significant comparisons (*p* < 0.05), differences among groups were analyzed using the Kruskal–Wallis test followed by Dunn’s post hoc test. Different letters indicate statistically significant differences (*p* < 0.05), whereas shared letters (*e.g.*, “ab”) indicate groups that are not significantly different. Data are presented as mean ± standard error of the mean (SEM) (n = 3).

To evaluate the biochemical stability of the recombinant endolysins, assays were performed specifically using *P. syringae* pv. *syringae* strain P63. LysX maintained stable lytic activity across the tested temperature range against this host, ranging from 36.9 ± 1.42% at 4 °C to 38.8 ± 1.95% at 18 °C. Slight reductions were observed at 25 °C (36.0 ± 2.76%) and 37 °C (36.4 ± 4.08%), while a more pronounced decrease occurred at 40 °C (18.7 ± 4.05%). However, this reduction did not reach statistical significance (Figure 5A). In contrast, LysP exhibited a marked temperature-dependent variation in lytic activity against the same host. The highest activity was observed at 4 °C (50.2 ± 12.4%) and 25 °C (49.2 ± 9.4%), followed by 18 °C (40.6 ± 4.55%). Activity decreased substantially at 37 °C (32.5 ± 1.7%) and dropped drastically at 40 °C (3.44 ± 2.0%), where the loss of activity was statistically significant compared to all other conditions (Figure 5B).

In the case of endolysin X, significant differences were also detected between pH 5 (19.5 ± 4.08%) and pH 9 (87.2 ± 6.07%), as well as between pH 8 (34.1 ± 2.89%) and pH 9, highlighting its enhanced activity under alkaline conditions (Figure 5C). At intermediate pH values, the activity was 44.4 ± 8.28% at pH 6, 38.8 ± 1.95% at pH 7, and 34.1 ± 2.89% at pH 8. For endolysin P, the highest activity was observed at pH 9 (61.6 ± 6.72%), with significant differences compared to pH 5 (19.5 ± 1.41%), as shown in Figure 5D. Intermediate values included 35.3 ± 9.68% at pH 6, 40.6 ± 4.55% at pH 7, and 27.1 ± 5.39% at pH 8, indicating a general trend of increased activity towards alkaline conditions, despite a slight decrease observed at pH 8.

## 3. Discussion

Analysis of the bacteriophage genomes for endolysin genes revealed that genome size does not correlate with the number of endolysin genes present. In the case of the genomic comparison of *X. arboricola* pv. *juglandis* bacteriophages, it was observed that, despite the similarity between these genomes, no relationship was observed with the number of genes identified as coding endolysins. The search for endolysins genes in *X. arboricola* pv. *juglandis* and *P. syringae* pv. *syringae* bacteriophage’s genomes was performed using different bioinformatics programs, complementing the information to effectively determine the required protein. Of the 25 identified genes, eight could not be assigned a putative function due to the strict adherence to selected parameters, even if some exhibited relevant functions. On the other hand, if these sequences of proteins were analyzed with other bioinformatics programs, it would be possible to find unique properties or determine that they belong to families that have not yet been characterized [34]. These sequences may represent novel lytic enzymes or members of uncharacterized families, underscoring the potential of phage genomes as a reservoir for discovering innovative antimicrobial tools. Of the 17 potential endolysins that met the parameters established for their identification, those that fulfilled the criteria described by Oliveira et al. [30], as well as on the concordance of their identified domains with hydrolytic activity, were one amidase family endolysin (LysP) and one glucosidase family endolysin (LysX). The antimicrobial potential of the enzymes was evaluated against *X. arboricola* pv. *juglandis* and *P. syringae* pv. *syringae*. Both endolysins demonstrated broad cross-genus activity, proving effective against both *Xanthomonas* and *Pseudomonas* strains. These results indicate that the recombinant endolysins possess lytic activity within or exceeding the ranges previously reported in the literature. Against *X. arboricola* pv. *juglandis*, the efficiency of LysP and LysX ranged from 51.5 ± 17.9% to 53.1 ± 11.3%, which is comparable to the efficiencies described by Wu et al. [35] against *Xanthomonas oryzae* pv. *oryzae* (19–74%). When tested against *P. syringae* pv. *syringae*, the observed efficiencies ranged from 62 ± 2.99% to 78.3 ± 1.26%, exceeding the values reported by Abdelrahman et al. [36] for *P. aeruginosa* (52.3%), and comparable to those described by Ni et al. [37] against *P. syringae* pv.* actinidiae* (68.1%). These findings indicate that these recombinant endolysins perform at a level equal to or even superior to those reported previously, supporting their potential as novel bactericidal agents.

The physicochemical characterization revealed important differences between the two enzymes. Endolysin P was more sensitive to elevated temperatures, showing a marked decrease in activity from 50.2 ± 12.4% to 3.44 ± 2.00%, corresponding to an approximately 14.6-fold reduction when incubated at 40 °C compared to lower temperatures. In contrast, endolysin X maintained higher stability across the tested temperature range. Both enzymes exhibited maximal lytic activity under alkaline conditions (pH 9), with activity levels exceeding 60% (X: 87.2 ± 6.72% and P: 61.6 ± 6.07%). Conversely, their lowest activity was observed under acidic conditions (pH 5), decreasing to values below 20% (X: 19.5 ± 4.08% and P: 19.5 ± 1.41%), representing an approximately four-fold reduction relative to activity at pH 9. These results are consistent with *in silico* analyses performed using the Compute pI/Mw (ProtParam) tool from the ExPASy Server [38], which predicted isoelectric points of 9.07 for LysP and 9.04 for LysX, suggesting that these enzymes are more stable and functionally active under conditions close to their theoretical pI.

These findings suggest that these recombinant endolysins perform at levels comparable to those reported previously, supporting their role as novel bactericidal candidates. Furthermore, their observed cross-genus activity against *Pseudomonas* and *Xanthomonas* highlights their potential as a versatile biocontrol strategy for addressing multiple economically significant plant pathogens in a commercial context.

Despite the promising antimicrobial properties observed, the large-scale application of purified endolysins is currently hindered by significant economic limitations, primarily the high costs associated with protein purification. In this study, purified enzymes were utilized to establish fundamental biochemical parameters; however, the use of non-purified lysates or enriched crude extracts represents a highly promising and cost-effective alternative for future field-scale applications. To this end, alternative production strategies must be explored. For instance, plant-based expression systems have emerged as efficient platforms for scalable protein production, offering a viable route for generating endolysin-enriched crude extracts for sustainable agricultural use [39,40,41].

In conclusion, the biochemical characterization and lytic efficiency demonstrated by LysX and LysP *in vitro* position these endolysins as promising candidates for the control of *P. syringae* and *X. arboricola*. However, it is recognized that the transition from laboratory assays to agricultural application requires evaluating these molecules in the complex environment of the plant phyllosphere. Future research will focus on *in planta* trials using detached leaves and fruits to assess how factors such as UV radiation, humidity, and leaf surface interactions may influence their stability and antimicrobial performance. This study provides the fundamental basis for developing new, sustainable biocontrol strategies against bacterial diseases in fruit trees.

## 4. Materials and Methods

### 4.1. Bacterial Isolates, Bacteriophages, and Growth Conditions

The characteristics of the bacterial isolates used in this work are summarized in Table 2. *P. syringae* pv. *syringae* isolate P63, *P. syringae* pv. *syringae* isolate S2, *X. arboricola* pv. *juglandis* strain 4.1 and *X. arboricola* pv. *juglandis* isolate 66a are wild-type isolates previously characterized in the Plant Biology and Agro-food Systems Innovation Laboratory (BVISA), INTA, University of Chile. All isolates were cultured in solid or liquid Luria–Bertani (LB) media (Thermo Fisher Scientific, Waltham, MA, USA). Phytopathogenic bacteria were incubated at 28 °C, whereas *Escherichia coli* was grown at 37 °C. Additionally, a total of 16 bacteriophages infecting *P. syringae* pv. *syringae* and *X. arboricola* pv. *juglandis* were used. These bacteriophages were isolated from environmental samples, such as irrigation canals and wastewater, between years 2015 and 2019, and form part of the collection available in the laboratory BVISA, INTA, University of Chile. Phage genomes were sequenced by an external service at Macrogen (Seoul, Republic of Korea).

### 4.2. Bioinformatics Analysis

The bacteriophage genomes were assembled and annotated using the bioinformatics programs Patric V3.33.16 [42] and Geneious V11.0.18+10 [43] for a preliminary characterization of genes encoding endolysins in each genome. The NCBI Basic Local Alignment Search Tool Protein (BLASTp) [44] was used to identify endolysin-coding sequences from the *P. syringae* pv. *syringae* and *X. arboricola* pv. *juglandis* phage genomes, using an identity value ≥ 75% and coverage ≥ 80% as parameters [30]. Furthermore, the presence of specific lytic domains was confirmed using InterPro v97.0 [31]. Putative endolysin genes were further classified into glycosidase (EC 3.2.1.17), amidase (EC 3.5.1.28), and endopeptidase (EC 3.4.16.-) families based on conserved domain analysis using InterPro and manual inspection of catalytic motifs. A comprehensive characterization of these genomic findings, including domain identifiers and sequence coverage for each identified gene, is presented in Supplementary Table S1. Predicted catalytic and binding residues were identified by aligning the deduced amino acid sequences with characterized endolysins from public databases.

The theoretical isoelectric point and molecular weight of the endolysins were determined using the Compute pI/Mw tool from the ExPASy server [38]. Additionally, AlphaFold V2.1.2 [32] and Swiss-Model [33] were employed to obtain two three-dimensional structural models. The secondary-structure content (number of α-helices and β-sheets) of the predicted models was assessed by structural inspection and compared across the generated models.

### 4.3. Vector Construction and Endolysin Expression

The endolysin LysX, encoded in the *X. arboricola* pv. *juglandis* phage 4.1 genome, was amplified by PCR using the primers Glu4.1F (5′-CGATCG**C**CATGGGAGGAAACCAGCAGCAGAACCAA-3′) and Glu4.1R (5′-ACCTTCTCGAGTCCTCCTCCCTTCACTTGGGGTTT-3′), which were designed in our laboratory. The forward primer contains an *Nco*I restriction site, while the reverse primer incorporates an *Xho*I restriction site. The resulting PCR product (586 bp) was purified and digested with *Nco*I and *Xho*I.

Similarly, the endolysin LysP, encoded in the *P. syringae* pv. *syringae* phage 63 genome, was amplified using primers Am63F (5′-CGATCGCCATGGGAGGAGCCAAGGTTCAATTCAA-3′) and Am63R (5′-ACCTTCTCGAGTCCTCCTCCGAGGCCGACCGTT-3′), which were designed in our laboratory. The resulting 489 bp PCR fragment was purified and digested with *Nco*I and *Xho*I.

Gene amplification was performed under an initial denaturation step at 98 °C for 30 s, followed by 30 cycles of denaturation at 98°C for 10 s and extension at 72 °C for 1 min, ending with an extension at 72 °C for 2 min. For amplification, the high-fidelity enzyme Q5^®^ High-Fidelity DNA Polymerase (New England Biolabs, Ipswich, MA, USA) was used.

The amplified genes were cloned into the expression vector pET21d (Novagen^®^, Sigma-Aldrich, St. Louis, MO, USA), and the insertion of the endolysin gene was verified using the universal primers T7 promoter and T7 terminator. The resulting pET21d-derived plasmids, containing the endolysin gene LysX or LysP, were denominated pLysX (5933 bp) and pLysP (5819 bp), respectively. These plasmids were independently transformed by thermal shock in the *E. coli* T7 Express *lys*Y strain (New England Biolabs, MA, USA) according to the manufacturer’s instructions. Plasmids containing the endolysin genes were sent to the Sequencing and Omics Technologies Unit of the Pontifical Catholic University of Chile for sequencing to verify both the insertion and the orientation of the genes.

The expression of the endolysin genes was carried out in exponential cultures (OD600 ≈ 0.5) of the transformed *E. coli* T7 Express *lys*Y strain. Protein expression was induced with 0.1 mM IPTG, and the cells were harvested after two hours of induction at 37 °C for endolysin LysP, and at 20 °C for endolysin LysX. Following cell disruption by ultrasonication, both endolysins were purified using the His-Spin Protein Miniprep™ kit (Symo Research, Irvine, CA, USA). The electrophoretic homogeneity and expected molecular weights for each purified fraction (21.8 kDa for LysX and 17.6 kDa for LysP) were independently verified by 15% SDS-PAGE. A negative control was included, in which the expression of the endolysins was not induced.

To halt bacterial growth, cultures were incubated on ice for 20 min, followed by centrifugation. The resulting pellet was resuspended in His-Binding buffer (50 mM sodium phosphate buffer pH 7.7; 300 mM sodium chloride; 10 mM imidazole; 0.03% Triton X-100) (Symo Research, USA) supplemented with 0.1 mM protease inhibitors. Cell disruption was performed using an ultrasonic cell disruptor (UCD-250 Series, BioBase, Jinan, China), applying 2 s pulses with 3 s intervals for a total of 5 min, while maintaining the samples on ice. Finally, the lysate was centrifuged at 10,000× *g* for 30 min at 4 °C, and the supernatant was collected and filtered through a 0.2 µm pore-size membrane filter. Endolysins were purified using the His-Spin Protein Miniprep™ kit (Symo Research, USA) based on the C-terminal 6xHis-tag. Total protein extracts from induced and non-induced *E. coli* cultures were analyzed by 15% SDS-PAGE alongside purified endolysin fractions. Samples were prepared by mixing with loading buffer and boiling for 5 min prior to electrophoresis. Protein concentrations were determined using the Qubit Protein BR Assay (Invitrogen™, Thermo Fisher Scientific, MA, USA).

### 4.4. Antimicrobial Activity Assay of Endolysin

Although CFU counting is a direct measure of viability, monitoring OD600 nm reduction is a widely accepted high-throughput method for initial endolysin characterization and kinetic analysis of cell wall degradation [45,46,47,48].

*P. syringae* pv. *syringae* S2 and *X. arboricola* pv. *juglandis* 66a bacterial cultures grew until reaching an OD600 of 0.4. To destabilize the bacterial membrane, cells were treated with 0.05% triton X-100 prior to the addition of endolysins. Bactericidal activity was evaluated by incubating the bacteria separately with each endolysin for 1 h at 25 °C. The lytic activity of the endolysins was determined using the formula described by Ding et al. [49]:

Relative lytic activity (%) = (OD_600 control − OD_600 endolysins)/(OD_600 control) × 100

For all lytic activity and characterization assays, purified endolysins were used at a final concentration of 10 µg/mL. Each experiment was performed in triplicate (n = 3) to ensure reproducibility and statistical robustness.

### 4.5. Characterization of Endolysin LysX and LysP

For the characterization of the endolysins, their stability under different pH and temperature conditions was evaluated. For pH stability, 50 mM Tris-HCl solutions adjusted to pH values ranging from 5 to 9 were prepared, and the endolysins were incubated with each buffer for 30 min at 4 °C. Similarly, for thermal stability, the endolysins were exposed to different temperatures (4, 18, 25, 37 and 40 °C) for 30 min in 50 mM Tris–HCl buffer at pH 7.0. In both assays, after the incubation period, the endolysins were incubated with the bacteria for 1 h at 25 °C, and their lytic activity was determined using the previously described formula. For thermal and pH stability assays, the lytic activity of endolysins was evaluated against *P. syringae* pv.* syringae* strain P63.

### 4.6. Statistical Analysis

The data did not meet the assumption of normality; non-parametric analyses were applied. Differences among groups were assessed using the Kruskal–Wallis test, followed by Dunn’s post hoc test with Bonferroni correction. Results were considered statistically significant at *p* < 0.05.

## 5. Conclusions

This study identified and characterized two recombinant endolysins, LysP and LysX, derived from phage genomes, which demonstrated promising antimicrobial activity against *X. arboricola* pv. *juglandis* and *P. syringae* pv. *syringae*. Both enzymes exhibited distinct physicochemical properties: LysX showed higher thermal stability and maintained activity under varying conditions, whereas LysP was more sensitive to high temperatures. Additionally, both endolysins displayed optimal lytic activity under alkaline conditions (pH 9) and reduced activity in acidic environments (pH 5), consistent with their predicted isoelectric points. When compared with previous studies, the efficiencies observed in this work were within or exceeded the range reported for other phage-derived endolysins, supporting their potential as effective bactericidal agents. Nevertheless, challenges remain regarding their large-scale application, mainly due to the high costs of protein purification. Alternative production platforms, such as plant-based expression systems, could provide viable and scalable solutions for implementation.

Overall, these findings contribute to the growing evidence supporting the application of phage endolysins as sustainable and environmentally friendly antimicrobial agents. Their demonstrated efficacy against key phytopathogenic bacteria highlights their potential as innovative tools for crop protection and reinforces the necessity of optimizing their stability and large-scale production. Moreover, the characterization of endolysins with distinct catalytic mechanisms—specifically the glycosidase LysX and the amidase LysP—facilitates the development of synergistic enzymatic cocktails. Such multi-target approaches could significantly enhance lytic potency while providing a robust strategy to mitigate the emergence of bacterial resistance in agricultural ecosystems.

## Figures and Tables

**Table 1:** Summary of hypothetical genes encoding different endolysin families in *P. syringae* pv. *syringae* and *X. arboricola* pv. *juglandis* bacteriophages.

	Glucosidase	Amidase	Endopeptidase	Unknown
Number of endolysin genes in phage *P. syringae* pv. *syringae*	6	4	0	2
Number of endolysin genes in phage *X. arboricola* pv.* juglandis*	6	0	1	6
Total number of genes	12	4	1	8

**Table 2:** Bacterial isolates used in this study.

Bacterial Isolate	Genotype	Source/Reference
T7 Express lysY competent *E. coli*	MiniF *lysY* (Cam ^R^)*/fhuA2 lacZ::T7 gene1 [lon] ompT gal sulA11 R(mcr-73::miniTn10–*Tet ^S^*)2 [dcm] R(zgb-210::Tn10–*Tet ^S^*) endA1 ∆(mcrC-mrr)114::IS10*	Commercially available (New England Biolabs).
*Pseudomonas syringae* pv.* syringae* S2	wildtype	This work
*Pseudomonas syringae* pv.* syringae* P63	wildtype	This work
*Xanthomonas arboricola* pv.* juglandis* 66a	wildtype	This work
*Xanthomonas arboricola* pv.* juglandis* 4.1	wildtype	This work

## Data Availability

The original contributions presented in this study are included in the article/Supplementary Materials. Further inquiries can be directed to the corresponding authors.

## Supplementary Materials

The following supporting information can be downloaded at: https://www.mdpi.com/article/10.3390/plants15030431/s1, Table S1: Identification and characterization of endolysins encoded in the genomes of *Pseudomonas syringae* pv. *syringae* and *Xanthomonas arboricola* pv. *juglandis* bacteriophages. The rows highlighted in blue are the endolysins encoded in the bacteriophages of *P. syringae* pv. *syringae* and *X. arboricola* pv. *juglandis* bacteriophages that were selected for subsequent expression.
