# Supplementary material for: Antimicrobial Activity of LysX and LysP Endolysins Against Pseudomonas syringae pv. syringae and Xanthomonas arboricola pv. juglandis
Source: Plants (Basel). 2026 Jan 30;15(3):431. doi: 10.3390/plants15030431 (PMC12899693; doi:10.3390/plants15030431)
Supplement: Supplementary file 1 [file plants-15-00431-s001.zip › plants-4089816-supplementary.pdf]

Supplementary Materials:

Table S1: Identification and characterization of endolysins encoded in the genomes of *Pseudomonas syringae* pv. *syringae* and *Xanthomonas arboricola* pv. *juglandis* bacteriophages. The rows highlighted in blue are the endolysins encoded in the bacteriophages of *P. syringae* pv. *syringae* and *X. arboricola* pv. *juglandis* bacteriophages that were selected for subsequent expression.

Phage	Function Found by Blastp	Amino Acid Identity	Coverage	E-value	Function Found by InterPro	Family	Domains	EC Number	Size endolysin (aa)
Xaj 4.1	Glucosidase	100% (Endolysin type T4)	100%	$5E^{-124}$	Glucosidase	Glucosidase Family 24 (IPR002196)	Lysozyme-like domain (IPR023346)	EC 3.2.1.17	185
Xaj 4.1	Transglycosylase lytic	96% (Transglycosylase SLT)	100%	0	Unknown	Unknown	Unknown	Unknown	1636
Xaj 6	Transglycosylase lytic	96% (Transglycosylase SLT)	100%	0	Unknown	Unknown	Unknown	Unknown	1636
Xaj 6	Glucosidase	100% (Endolysin type T4)	100%	$5E^{-124}$	Glucosidase	Glucosidase Family 24 (IPR002196)	Lysozyme-like domain (IPR023346)	EC 3.2.1.17	185
Xaj 36	Transglycosylase lytic	96% (Transglycosylase SLT)	100%	$3E^{-88}$	Glucosidase	Unknown	Transglycosylase SLT domain 1 (IPR008258)	EC 3.2.1.17	149
Xaj 36	Glucosidase	100% (Transglycosylase SLT)	100%	$5E^{-124}$	Unknown	Unknown	Signal peptide	Unknown	185
Xaj 36	Transglycosylase lytic	96% (Transglycosylase SLT)	100%	0	Unknown	Unknown	Unknown	Unknown	1512
Xaj M2-2	Transglycosylase lytic	96% (Transglycosylase SLT)	100%	0	Unknown	Unknown	Unknown	Unknown	1636
Xaj M2-2	Glucosidase	100% (Endolysin type T4)	100%	$5E^{-124}$	Glucosidase	Glucosidase Family 24 (IPR002196)	Lysozyme-like domain (IPR023346)	EC 3.2.1.17	185
Xaj M4-109a	Transglycosylase lytic	96% (Transglycosylase SLT)	100%	0	Unknown	Unknown	Unknown	Unknown	1636
Xaj M307-70a	Glucosidase	59% (Chitinase)	78%	$4E^{-100}$	Glucosidase	Glucosidase Family 19 (IPR000726)	Chitinase domain (PTHR22595)	EC 3.2.1.14	233
Xaj M307-70a	Cell wall hydrolase	59% (Hydrolase SleB)	91%	$9E^{-66}$	Glucosidase	Cell wall hydrolase, SleB (IPR011105)	SleB domain 1 (IPR042047)	Unknown	184
Xaj M310-91a	Endolysin	63% (Endopeptidase)	99%	$9E^{-53}$	Endopeptidase	Unknown	L-alanyl-D-glutamate endopeptidase (cd14845)	EC 3.4.16.	127

Pss 7	N-acetylmuramoyl-L-alanine amidase	100% (Endolysin type T7)	100%	$8E^{-106}$	Amidase	Endolysin type T7 family (IPR034689)	Peptidoglycan recognition domain and N-acetylmuramoyl-L-alanine amidase domain (IPR036505)	EC 3.5.1.28	147
Pss 11	Glucosida se	55% (Hydrolase TtsA)	94%	$8E^{-58}$	Glucosidase	Glucosidase Family 108 (PF05838)	TtsA-like glycosyl hydrolase 108 domain (IPR008565)	EC 3.2.1.17	191
Pss 12	N-acetylmuramoyl-L-alanine amidase	100% (Endolysin type T7)	100%	$8E^{-106}$	Amidase	Endolysin type T7 family (IPR034689)	Peptidoglycan recognition domain and N-acetylmuramoyl-L-alanine amidase domain (IPR036505)	EC 3.5.1.28	147
Pss 22	Transglycosylase lytic	57% (Transglycosylase SLT)	89%	$1E^{-64}$	Unknown	Unknown	Unknown	Unknown	200
Pss 25	Transglycosylase lytic	57% (Transglycosylase SLT)	89%	$1E^{-64}$	Unknown	Unknown	Unknown	Unknown	200
Pss 43	Lisozima	82% (Lysozyme type)	99%	$7E^{-107}$	Glucosidase	Unknown	N-acetylmuramidase (IPR024408)	EC 3.2.1.17	185
Pss 56a	N-acetylmuramoyl-L-alanine amidase	100% (Endolysin type T7)	100%	$1E^{-109}$	Amidase	Endolysin type T7 family (IPR034689)	Peptidoglycan recognition domain and N-acetylmuramoyl-L-alanine amidase domain (IPR036505)	EC 3.5.1.28	153
Pss 56a	Lysozyme	96% (Lysozyme type)	100%	0	Glucosidase	Unknown	N-acetylmuramidase (IPR024408) and Peptidoglycan-binding-like domain (IPR002477)	EC 3.2.1.17	281
Pss 58	Lysozyme	82% (Lysozyme type)	99%	$7E^{-107}$	Glucosidase	Unknown	Glucosidase domain	EC 3.2.1.17	185
Pss 58	Lysozyme	96% (Lysozyme type)	100%	0	Glucosidase	Unknown	N-acetylmuramidase (IPR024408) and Peptidoglycan-binding-like domain (IPR002477)	EC 3.2.1.17	281

Pss 63	N-acetylmuramoyl-L-alanine amidase	100% (Endolysin type T7)	100%	$8E^{-106}$	Amidase	Endolysin type T7 family (IPR034689)	Peptidoglycan recognition domain and N-acetylmuramoyl-L-alanine amidase domain (IPR036505)	EC 3.5.1.28	147
Pss 63	Glucosidase	100% (Endolysin type T4)	100%	$5E^{-124}$	Glucosidase	Glucosidase Family 24 (IPR002196)	Lysozyme-like domain (IPR023346)	EC 3.2.1.17	185
